# Evaluating the concordance of physician judgments and patient preferences on AIDS/HIV therapy - a Discrete Choice Experiment

**DOI:** 10.1186/2191-1991-3-30

**Published:** 2013-12-18

**Authors:** Axel C Mühlbacher, Matthias Stoll, Jörg Mahlich, Matthias Nübling

**Affiliations:** 1 Hochschule Neubrandenburg, Brodaer Straße 2, Neubrandenburg 17033, Germany; 2 Gesellschaft für empirische Beratung, Empirical Consulting, Denzlingen, Germany; 3 Medizinische Hochschule Hannover, Hannover, Germany; 4 Janssen Cilag, Neuss, Germany; 5 University of Vienna, Vienna, Austria

**Keywords:** AIDS/HIV, Physician judgment, Patient preferences, Stated preferences, Discrete-choice-experiment (DCE), Direct assessment

## Abstract

**Objectives:**

Patient-centered health care and shared decision making are of increasing importance in the management of AIDS/HIV patients and require an intensive consideration of patient preferences. The present study assesses expectations and needs of patients from the physician point of view. The aim of this study was to compare patient and physician perspectives of relevant aspects of treatment quality such as effectiveness, quality of life and further treatment options.

**Methods:**

The study was performed as an anonymous survey including German physicians. Physicians treating large numbers of AIDS/HIV patients were preferably contacted. The physicians were asked to assess their view of patient preferences of therapy characteristics using direct measurement, as well as by means of a Discrete Choice Experiment (DCE). The questionnaire was adopted from a previous study in which AIDS/HIV patients were asked to assess their treatment preferences.

**Results:**

131 physicians completed the questionnaire, 88% of these on paper and 12% online. 70% of the physicians were male. The mean duration since licensure was 17 years. The most frequent specialist areas were internal medicine (N = 55), infectiology (N = 31) and general medicine (N = 27). In the direct measurement the most relevant therapy characteristics were “drug does not affect or not affect appearance much”, “self-application of the drug is possible” and “rarely occurring longer periods of nausea and diarrhea”. Six treatment characteristics were selected and used to generate eight virtual pairs of therapies. To evaluate the assessments a random effect logit model was employed. In view of the physicians avoidance of an obvious perceptibility of the disease the emotional quality of life had by far the strongest impact on the patients’ treatment preferences as rated by physicians. With some distance the physical quality of life with less diarrhea or nausea, as well as the possibility to participate in social life followed on the same level.

**Conclusions:**

Discrete Choice Experiment proved to be a valid survey technique in the evaluation of AIDS/HIV treatment preferences as assessed by patients and by physicians assessing the view of their patients. Covering a broad range of treatment characteristics, the physician assessments of preferences were very close to those of AIDS/HIV patients emphasizing the high impact of quality of life, in particular the emotional quality of life on patient preferences in the selection of treatments. Thus, the selection of particular treatment options should be accompanied by a deliberate consideration of treatment features, which need to be considered in order to maximize patient adherence and compliance.

## Background

The acquired immune deficiency syndrome (AIDS) is a disease with failure of the immune system caused by the human immune deficiency virus (HIV). It is characterized by certain life-threatening opportunistic infections and malignancies. Progression to AIDS can be delayed by combined antiretroviral therapy (cART, [1]). HIV-infection occurs by mucosal or parenteral body fluid contact, i.e. blood, semen, vaginal fluid, pre-ejaculate, and breast milk [2]. AIDS was first reported in 1981 [3]. At the end of 2011, an estimated 34 million people were living with HIV worldwide. Hence, HIV infection in humans is classified as pandemic by the World Health Organization [2]. The number of people dying of AIDS-related causes dropped to 1.8 million in 2010, down from a peak of 2.2 million in the mid-2000s. In 2011 there were 2.5 million new HIV infections. This was 20% less than in 2001. According to UNAIDS data Germany has an estimated HIV prevalence of 0.1. In 2011 approximately 73,000 Germans were living with HIV/AIDS [4].

The availability of cART that reduces both the mortality and morbidity associated with HIV infection is one of the main factors influencing this decrease [2]. In addition the effective reduction of viral load through cART substantially reduces the risk of HIV transmission [5]. HIV viral suppression, reduced rates of resistance, an increase in survival*,* and improved quality of life have shown to be strongly correlated with adherence to antiretroviral therapy [6,7]. Besides quality of life the dosing is being discussed as patient-relevant. Because HIV treatment is a lifelong endeavor, and because many patients will initiate therapy when they are generally in good health without obvious signs or symptoms of HIV disease, adherence poses a special challenge depending on the side-effects and requires commitment from the patient and the health care team [8].

Understanding about patient preferences characterized through expectations and needs concerning therapeutic options is an important prerequisite to understand patient adherence. Research on this topic is of increasing interest and supplements evidence-based results on efficacy and tolerability. As an important part of benefit-risk assessments the evaluation of patient preferences is recommended by the American FDA [9], as well as by the German institutions G-BA (Federal Joint Committee) [10] and IQWIG (Institute for Quality and Efficiency in Health Care), which explicitly encourage the integration of conjoint analyses of patient preferences [11].

In a previous study [12] we evaluated therapy-related expectations and needs of AIDS/HIV-patients using direct measurement, as well as by means of a Discrete Choice Experiment (DCE). 218 patients completed this study. In the direct measurement the most relevant therapy characteristics were “Self-application of the drug (at home or on-the-go) possible”, “Drug has very high efficacy (reduction of viral load)” and “Long term (hidden) side-effects (e.g. organ damage) is unlikely”. Based on these data eight hypothetical therapy scenarios were generated and used in a Discrete Choice Experiment. The main result was the high impact of quality of life, in particular the emotional quality of life on patient preferences on the selection of treatments.

The present study assesses expectations and needs of AIDS/HIV-patients from the physician point of view, however, not reflecting their own preferences, but their view of patient preferences. The aim of this study was to assess physicians’ judgments of relevant aspects of treatment quality such as effectiveness, quality of life and further treatment options. In a second step these results should be compared to the findings on patient preferences. This objective gets even more important on the background of increased patient-centered healthcare and the postulation of shared decision making among health professionals and patients. For that reason an understanding of the concordance of physicians and patients is absolutely essential. The study procedure has already been established in the therapy of multiple myeloma [13], where some congruency between patient and physician assessments was found, although physician and patient communication has been shown to be critical to determine the best treatment options regarding patient preferences.

## Methods

The study was performed as an anonymous survey including German physicians. Data were collected from October until November 2010 using either online or paper questionnaires. The physicians were contacted either in writing and were given the paper-based questionnaire version with stamped addressed envelopes, or via email/internet. Both methods were offered since older physicians may not have wanted to use the online version. In terms of content, there were no differences between both versions of the questionnaire. In recent studies both methods of administration have proven to be highly reliable and comparable [14].

The recruitment of physicians was performed using a nationwide database from Janssen-Cilag GmbH attended for informational and marketing purposes. From this database those physicians were selected who were known for treating large numbers of AIDS/HIV patients, because it was expected that these physicians are very experienced concerning patient preferences. A total of N = 404 physicians were contacted. Both paper-based questionnaires and the link to the online version were sent to the selected physicians. No personal data such as addresses, names or phone numbers were collected. It was intended to get a return of 100 evaluable questionnaires as this number would be sufficient for DCE-estimates according to the formula for sample size calculation by Johnson and Orme [15]. Moreover a sample size of N = 100 would have been representative of the HIV specialists (e.g. general practitioners, venereologists, internists) in Germany.

The questionnaire encompassed the following sections:

○ Section A (physician characteristics): socio-demographic parameters of physicians: gender, number of years in the profession, specialization, self-assessment of knowledge about patient preferences.

○ Section B (practice characteristics): Frequency of AIDS/HIV patients in practice, frequencies of treated and untreated patients, frequencies of patients being experienced or naïve about treatment.

○ Section C: Direct assessment of importance of 26 items on AIDS/HIV therapy characteristics (Five-point Likert scale).

○ Section D: Discrete Choice Experiment (DCE) for patient preferences from the physician point of view using eight pairs with six characteristics each.

The 26 items on AIDS/HIV therapy characteristics of section C (shown in Table 1) and the eight pairs of treatment comparisons of section D were adopted from a previous study [12] in which AIDS/HIV patients were asked to assess their treatment preferences. In this study, treatment characteristics had been developed using a qualitative pre-study. Furthermore, summarizing aspects of importance of treatment characteristics had been identified by factor analysis and pair comparisons for the Discrete Choice Experiment had been generated by an orthogonal experimental design [12].

**Table 1:** Mean importance of therapy characteristics assessed by physicians taking the view of their patients and by the patients themselves

**Mean importance**		
**Physicians**^**1**^	**Patients**^**2**^	**Therapy characteristic**
*Benefits*		
85	89	Drug improves physical state (e.g. better mobility, pain relief)
84	94	Drug has very high efficacy (reduction of viral load)
**83**	**90**	**Drug promises maximum prolongation of life expectancy**
**77**	**81**	**Drug improves emotional and mental state (e.g. less thoughts about disease)**
76	82	Drug allows for improved mobility (e.g. longer journeys possible)
**76**	**78**	**Drug improves social contact opportunities (e.g. visits possible)**
73	85	Long duration of efficacy following application
62	88	Drug allows further therapy options (in the future)
60	90	Drug does not generate resistance
*Side effects*		
92	89	Drug does not or does not affect appearance much (disease not visible, e.g. by fat redistribution)
**87**	**86**	**Rarely occurring longer periods of nausea due to drug application**
**87**	**86**	**Rarely occurring longer periods of diarrhea due to drug application**
**79**	**92**	**Long term (hidden) side-effects (e.g. organ damage) is unlikely**
57	30	Pregnancy is allowed during treatment period
56	80	Drug can be used also in case of comorbidities
*Mode of administration*		
90	94	Self-application of the drug (at home or on-the-go) is possible
86	82	Flexible application during course of the day (e.g. delay of 1 hour is possible)
**86**	**79**	**Simple application: only few tablets**
83	85	Drug can be taken along without problems (transportation)
81	73	Inconspicuous drug intake (discreet, unnoticed by environment)
77	73	Drug has to be taken only once daily
76	84	Long term use of the drug is possible
69	75	Drug does not cause additional costs for patient (no extra payment)
68	71	Treatment does not require much time (waiting time, time for treatment)
47	51	Therapy-free intervals/treatment breaks are possible
35	60	Dosing of drug may vary according to current health state

^**1**^Results of the present study, ^**2**^Results of the previous study [12], bold: attribute used in DCE.

Ethical considerations: The study is a social science survey and does not contain personal data (completely anonymous survey), surgeries (tests, experiments, and medication), biomedical research or additional data, as in many epidemiological investigations. Therefore an ethic vote in Germany was not necessary. All respondents were informed about the study and its potential risks and benefits prior the participation. Respondents had to sign an informed consent. They participated voluntarily and the participant could stop at any time. The study with all information material and the survey instrument was approved by the Janssen-Cilag GmbH.

### Conjoint analysis and Discrete Choice Experiment (DCE)

The Discrete Choice Experiment is a choice based method, and a variant of the conjoint analysis that was made possible through the theoretical work of Lancaster [16] and McFadden [17]. In the Discrete Choice Experiment different therapies are presented pair-wise and the subjects have to decide for one of the options [18]. In a first step all characteristics that are relevant for each target group have to be identified [19].

The treatment alternatives are presented to the subject and the subject has to decide for one of the presented options. Based on the decision behavior the relevance of the different characteristics for the decision can be calculated and described by coefficients. The calculation of coefficients is performed via the maximum likelihood method. According to the underlying distribution function different estimation methods are applied. In most cases these are probit or logit estimations [20-22].

To appreciate the importance of possible statistical correlations between main effects and interactions, the number of combinations was reduced to a more manageable size without losing essential information through an orthogonal design (when making certain assumptions about interaction effects). The SPEED software package was used to select the optimal subset of scenarios [23], maximum dissimilarity between therapy alternatives was achieved by generating the alternative B as exact mirror image of alternative A (using the fold-over technique) [12]. In this study we conducted the DCE technique with eight pair decisions, each with six characteristics. Respondents had to choose eight times between treatment A or B. The presented treatment pairs, as well as the characteristics were the same as in the previous patient study with verbal adaptions to physicians. Linguistically, the questions were adapted in the questionnaire for physicians from a self-assessment: “What would you choose …?” into a judgment: “How do you think your patients would rate” and “What would your patient choose?”.

### Statistical analysis

For statistical data analysis we used analysis of variance, regression analysis, factor analysis, and random effect logit models for the DCE. All statistical analyses were done using SPSS and STATA. A p-value <0.05 (two sided) was considered as being statistically significant.

Direct assessments of the importance of therapy characteristics were evaluated for effects of socio-demographic baseline characteristics of the physicians using analyses of variance. However, the problem of multiple testing has to be borne in mind: with more than 200 statistical tests to be performed some significant results will be produced “by chance”, they are however artefacts (approx. 10 of such results must be expected at a 95% confidence level). A possible increase of significance level (e.g. Bonferroni correction) may minimize these errors but increases the risk of ignoring really existing relationships. Therefore, a significance level of p < 0.05 was used.

## Results

### Physician characteristics

Between October and November 2010, N = 131 physicians completed the questionnaire. Most of the physicians (88%) answered via paper version and 12% online. 70% of the physicians were male.

The year of licensure is used as an indicator for professional experience. 33% of the physicians were practicing for 20 years or more, 46% for 10 to 20 years and 21% had a professional experience of less than 10 years. The mean duration of licensure was 17 years (1993). 82% of the physicians were specialists in at least one field with a maximum of five fields. The most frequently mentioned specialties were internal medicine (N = 55), infectiology (N = 31) and general medicine (N = 27).

A summary of physician characteristics is shown in Table 2.

**Table 2:** Physician characteristics, professional experience and knowledge of preferences

**Parameter**	**Actual data**
N	131 physicians
Gender	
Male	70%
Female	30%
Completion of survey	
Paper and pencil	88%
Online	12%
Year of licensure	
Mean	1993
1990 or earlier	33%
1991-1999	46%
2000 or later	21%
Knowledge about patient preferences	
Very good	34%
Good	60%
Medium	5%
Not so good	1%

34% of the physicians claimed to have “very good” knowledge about patient preferences; 60% rated their level of knowledge as “good”. A total of 5% thought they had a “medium” level of knowledge about preferences, whereas 1% claimed it was “not so good”.

### Practice characteristics

As the questionnaires were sent preferably to physicians known for large patient numbers, 85% of the physicians documented that they had treated 50 AIDS/HIV patients or more during the last 12 months. 8% had treated 25–49 patients, 3% had treated 10–24 patients and 3% had treated 1–9 patients. A small proportion of physicians (1.5%) had not treated or counseled any AIDS/HIV patients in the previous 12-month period.

66% of the physicians documented that at least 75% of their AIDS/HIV patients received antiretroviral treatment and 28%, 5% and 2% of the physicians documented that 50-74%, 25-49% and 0-24% of their AIDS/HIV patients received antiretroviral treatment, respectively.

### Direct assessment of AIDS therapy characteristics

Specific characteristics that patients consider relevant in the assessment of antiretroviral treatments were collected on the basis of the previous study [12]. The physicians had to rate the importance of 26 therapy characteristics from their patients’ point of view using a Five-point Likert scale, ranging from “very important” to “not important”. Specific point values of 0 (“not important”) to 100 (“very important”) were equally attached to the five Likert categories. The mean assessments of importance are shown in Table 1, which also contains the patient assessments resulting from the previous study [12].

Most of the items concerning quality of treatment are believed to be of relatively high mean importance (>70 points) for the patients as assessed by the physicians. These ceiling effects are not surprising, since only aspects were presented, that were rated as important according to the literature and the qualitative pre-study during the previous patient preference study.

### Influence of socio-demographic characteristics of physicians

Using analyses of variance, the assessments were evaluated for effects of socio-demographic baseline characteristics of the physicians. The results showed in particular that physicians with more professional experience and physicians with a higher number of treated patients assessed some characteristics as more important than the other physicians:

Physicians with more professional experience assessed three items as significantly more important: “Drug has very high efficacy”, “Drug does not generate resistance”, “Long term side-effects are unlikely”.

Physicians with large patient numbers assessed six items as significantly more important: “Inconspicuous drug intake”, “Rarely occurring longer periods of diarrhea”, “Long term side-effects are unlikely”, “Drug improves physical state”, “Drug does not affect or does not affect appearance much” as well as “Drug improves emotional and mental state”.

### Differences between physician and patient assessments

In the previous study asking patients as well as in the present study asking physicians the same 26 items were used to assess the relevance of treatment characteristics from the view of the patients, thus allowing a comparison between the assessments of patients and physicians. In most of the aspects the assessments of the physicians were close to those of the patients – the differences between both mean values were mostly below 10 points. This indicates that physicians had a good feeling for the desires and needs of the patients in most of the aspects.

Differences were found for the item “Pregnancy is possible”, which was assessed as much more important by physicians as compared to patients; however, this item was not particularly important in both groups, which implicates that the different assessments of relevance are both in the lower range of importance. When comparing both data sets it becomes obvious that physicians made clearly lower relevance assessments on four aspects than the patients did. These were the items “Further treatment options possible”, “No resistances generated”, “Drug can be used also in case of comorbidities” and “Dosing may vary according to current health state”. Obviously, the physicians underestimated the relevance of these aspects as assessed by the patients. Interestingly, these differences between physicians and patients were only found in aspects that were not different in subgroups of physicians, implicating that the misinterpretations of patient assessments occurred with all physicians, not only with some of them.

### Preferences in the Discrete Choice Experiment

Discrete Choice Experiments (DCE) are limited to the use of only a few characteristics. Based on the previous study on patient preferences [12] using the same 26 therapy characteristics and a following principal components analysis (PCA) including varimax rotation, six characteristics were selected and described by a positive and a negative pole (see Table 3).

**Table 3:** Treatment characteristics for Discrete Choice experiment

**Characteristics**	**Positive pole (+)**	**Negative pole (-)**
Life expectancy	Maximal increase	Moderate increase
Long term side-effects	Improbable (<20% of patients)	Possible (≥20% of patients)
Flexibility of dosing	Max. 3 tablets/day	≥4 tablets/day
Physical quality of life	Diarrhea, nausea less frequent	Diarrhea, nausea more frequent
Emotional quality of life	Disease not obvious for others	Disease obvious for others
Social quality of life	Participation in social life possible	Participation in social life restricted

### Generation of pairs

Based on the six therapy characteristics eight virtual therapies were generated. These eight therapies were presented to the patients in eight pairs from which the patient had to select one of the two therapies (A or B).

In total, 1047 valid assessments were available from 131 physicians. As each physician had to make 8 assessments, only one assessment was missing.

A random effect logit model was created, which takes the partial dependency of observations from the same person concerning the parameter estimation into account. Estimated coefficients and their standard errors are shown in Table 4.

**Table 4:** Results of random effect logit model (Discrete Choice Experiment; negative pole as reference group)

**Characteristics**	**Coefficient**	**SE**	**Sig.**	**Partial log**
		**(coeff.)**		**likelihood**
Life expectancy: maximum increased	0.846	0.265	**	-373.0
Long term side-effects improbable (<20%)	0.831	0.224	***	-374.5
Flexibility of dosing: max. 3 tablets/day	0.635	0.261	*	-370.3
Physical quality of life: diarrhea, nausea less frequent	1.925	0.256	***	-402.7
Emotional quality of life: disease not obvious for others	4.003	0.269	***	-604.8
Social quality of life: participation in social life possible	1.947	0.269	***	-402.1
Model constant	-5.316	0.417	***	

***p < 0.001, **p < 0.01, *p < 0.05.

Model parameters:

LR Chi^2^ (df = 6) = 715.90.

Log likelihood = -367.7.

Prob > Chi^2^ = 0.0000 (i.e. p < 0.001, ***).

% correctly classified = 85.0%.

In this model significant (non-zero) estimations for the positive poles were demonstrated for all six characteristics indicating the expected direction: the positive pole was chosen significantly more frequent than the negative one. Thus, all six parameters are statistically significant predictors of treatment preferences.

As shown in Table 4, the patient treatment preferences assessed by the physicians depend on the six characteristics to a different degree. From the physicians point of view avoidance of an obvious perceptibility of the disease (“Emotional quality of life”; characteristic 5) had by far the strongest impact on the patient treatment preferences. With some distance the “Physical quality of life” (characteristic 4) with less diarrhea or nausea, as well as the “Possibility to participate in social life” (characteristic 6) followed on the same level. With a clear distance but on the same level “Maximum increased life expectancy” (characteristic 1) and “Avoidance of long-term side-effects” (characteristic 2) followed. In the view of the physicians the “Flexibility of dosing”, represented by an intake of a maximum of 3 tablets per day (characteristic 3) had the lowest but still statistically significant impact.

Differences between the attribute “Emotional quality of life” (characteristic 5) and each of the other five characteristics were statistically significant at least on a p < 0.05 level. Thus, the relevance of this treatment characteristic for treatment preferences is significantly higher than that of every other characteristic. The characteristics “Physical quality of life” and “Social quality of life” were not significantly different in relevance on treatment preferences. However, both were significantly different from each of the other four characteristics: both were less relevant than characteristic 5, but more relevant than each of the characteristics 1, 2 and 3. No significant differences were found between characteristics 1, 2 and 3, but each of them is less relevant on treatment preferences than the characteristics 4, 5 and 6.

A supplementary partial log likelihood analysis as proposed by Lancsar et al. [24] yielded a similar hierarchy as the interpretation based on the item coefficients.

In additional interaction analyses, subgroup effects were found on a bivariate level for the characteristics “Maximum increased life expectancy”, “Avoidance of long-term side-effects”, “Physical quality of life” as well as “Emotional quality of life”. Using a simplified multivariate model, including only significant subgroup effects in addition to main effects, three effects were found:

• Physicians without specialization assessed the relevance of “Maximum increase of life expectancy” (characteristic 1) higher than their colleagues with specialization.

• In contrast, they assessed the relevance of “Emotional quality of life” (characteristic 5) lower than their colleagues with specializations.

• Physicians who documented having a very good knowledge of patient preferences assessed a low relevance for “Long-term side-effects improbable” (characteristic 2).

### Differences between physician and patient preferences

The Discrete Choice Experiment assessments of this study present the physician view about what they think the preferences of patients are.

The total set of treatment comparisons including physician and patient preferences was adopted from Mühlbacher et al. [12]. Both physician and patient preferences correspond to a high degree. They show rather clear decisions in five of the eight pairs, whereas in three pairs the less frequently chosen alternative was selected in at least 22% of the assessments.

**Figure 1:**
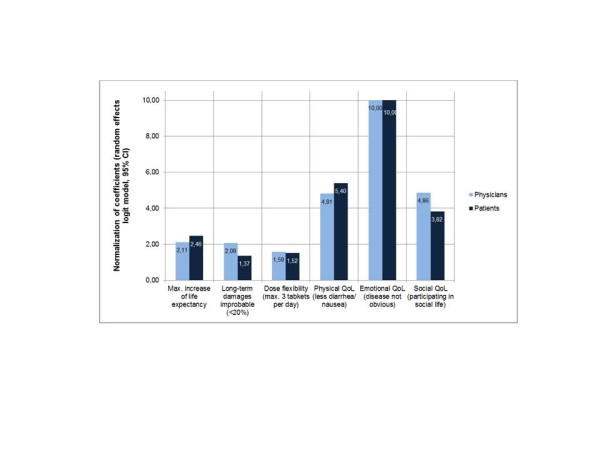
**Coefficients of Discrete Choice Experiment (positive pole) for physicians (data of present study) and patients (data of previous study,**[12]**) (normalization of coefficients).**

The patient preferences assessed by the patients themselves had been collected in a previous study [12] using the same study material. The DCE results of both studies are presented in Figure 1, while Table 5 displays the results of random effects logit models for both groups.

**Table 5:** Results of random effect logit model (Discrete Choice Experiment; negative pole as reference group; patients vs. physicians)

**Characteristics**	**Patients**^**1**^				**Physicians**^**2**^			
	**Coeffi-cient**	**SE (coeff.)**	**Sig.**	**Partial log likelihood**	**Coeffi-cient**	**SE (coeff.)**	**Sig.**	**Partial log likelihood**
Life expectancy: maximal increase	0.735	0.152	***	-717.7	0.846	0.265	**	-373.0
Long term side effects improbable (<20%)	0.408	0.147	**	-709.5	0.831	0.224	***	-374.5
Flexibility of dosing: max. 3 tablets/day	0.454	0.151	**	-710.1	0.635	0.261	*	-370.3
Physical quality of life: diarrhoea, nausea less frequent	1.611	0.152	***	-769.4	1.925	0.256	***	-402.7
Emotional quality of life: disease not obvious for others	2.984	0.153	***	-981.7	4.003	0.269	***	-604.8
Social quality of life: participation in social life possible	1.140	0.153	***	-735.4	1.947	0.269	***	-402.1
Model constant	-3.726	0.214	***		-5.316	0.417	***	

^1)^ ***p < 0.001, **p < 0.01, Model parameters: Wald Chi2 (df = 6) = 470.81, Log likelihood = -705.7, Prob > Chi2 = 0.0000 (i.e. p < 0.001, ***), Prob ≥ Chi2 = 1.000.

^2)^ ***p < 0.001, **p < 0.01, *p < 0.05; Model parameters: LR Chi^2^ (df = 6) = 715.90, Log likelihood = -367.7, Prob > Chi^2^ = 0.0000 (i.e. p < 0.001, ***), % correctly classified = 85.0%.

Respondents’ choices are made conditional upon the levels presented in the choice set. Choices inform about respondents perceived (expected) utility of each alternative within the choice scenarios. The mean relative importance of the attributes can be interpreted as the difference between the upper and the lower level.

As the mean relative importance is measured on an arbitrary scale, we have normalized the most important attribute (highest coefficient as illustrated in Table 5) as 10 units and measured the other attributes’ importance relative to this change (illustrated in Figure 1). Instead of analyzing mean relative importance, trade-offs between changes in time or cost can be explicitly mapped. Measuring people’s willingness to make trade-offs is consistent with welfare economics. It can be measured by determining the disutility which is generated through a change from the most preferred level to the least preferred level and by identifying the corresponding increase in healthy life years, or decrease in cost that is needed to return to the original level of utility. Since we did not include cost or time, we were not able to estimate money equivalents or healthy-years equivalents.

Two aspects are particularly striking:

• The ranking order of the six characteristics is nearly identical for both groups. By far most important is the characteristic “Emotional quality of life”, followed by “Physical quality of life” and “Social quality of life” (on the same level in physicians; with some difference in patients) and the characteristics “Maximum increased life expectancy”, “Long-term side-effects improbable” and “Flexibility of dosing” follow on rank 4 to 6.

• The most important differences between both groups are:

○ a) Patients assessed the characteristic “Less diarrhea/nausea” higher than “Participating in social activities is possible” and

○ b) Patients assessed the “Maximum increase of life expectancy” somewhat lower than physicians whereas physicians assessed the “Avoidance of long-term side-effects” much higher than patients themselves.

At the end of the questionnaire, the physicians were asked to give an explicit additional assessment of the degree of difficulty they had in performing the paired comparisons in the DCE. 2% assessed the ratings as “very difficult”, 26% said it was “rather difficult”, 43% gave a medium ranking (“some comparisons more difficult than others”), 24% thought it was “not very difficult” and 5% claimed they had “no problems at all” in completing the DCE. This means that the DCE can be considered as feasible from the physician point of view.

## Discussion

Patient opinions and desires concerning medical services (e.g. medication, therapy modalities) are often not sufficiently considered. However, taking into account concepts like “patient involvement” and “shared decision making”, an understanding of patient priorities concerning treatment decision making is required. Furthermore, it is important to evaluate whether the attending physicians sufficiently know the preferences of their patients to consider them adequately in (mutual) treatment decisions.

In a previous study (N = 218), we analyzed patient preferences in the treatment of AIDS/HIV using direct assessments and a Discrete Choice Experiment [12]. The study presented here used the same assessments for a survey among physicians (N = 131), however, asking not about their own preferences, but about their view of patient preferences. Subsequently, a Discrete Choice Experiment was used to determine the degree to which each therapy characteristic influenced the decisions.

The survey was offered paper-based and as online questionnaire. The intense usage of the paper version was probably due to the recruitment procedure, which included sending off the paper-based questionnaire. As the recruitment concentrated on physicians known for large numbers of AIDS/HIV patients, the study results can be seen as being representative only for this group of physicians. However, it was supposed that physicians experienced with treating a large proportion of HIV/AIDS-patient are also knowledgeable to patient needs and priorities. The best, albeit most demanding, approach would be to ask physicians and their (real) patients at the same time. However, this was not possible within the scope of this study. In order to achieve highest possible comparability we recruited highly experienced physicians that treat a large number of HIV/AIDS patients. Moreover, HIV/AIDS patients are highly active and well organized in terms of self-help groups. They are highly informed and often have long-term relationships with their physicians, which enables shared-decision making as well as a good judgment by physicians.

For the direct assessment of preferences physicians had to assess 26 treatment characteristics which had been found to be relevant in the previous study [12] based on literature research and a qualitative pre-study. As expected, a ceiling effect was found with ratings in the upper range of the scales for many of the characteristics. This might be due to the fact that, resulting from the qualitative pre-study, mainly important characteristics were presented. Within the rating, the top of the priority list is marked by preferences such as “drug does not affect or does not much affect appearance”, “self-application is possible”, but also by avoidance of side effects.

In most of the treatment characteristics the assessments of physicians seen in this study and patients found in the previous study [12] are congruent. In some characteristics, however, there are obvious differences: thus, compared to patient assessments, physicians tend to underestimate the relevance of the characteristics “Drug allows further therapy options in the future”, “Drug does not generate resistance”, “Drug can be used also in case of comorbidities” and “Dosing of drug may vary to current state”. Effects of socio-demographic baseline characteristics of the physicians showed that professionally more experienced physicians assessed “High efficacy”, “Not generating resistance” and “Unlikely long term side-effects” as being more important, while physicians with large numbers of patients for example assessed “Unlikely long term side-effects”, “Improvement of physical state” and of “Emotional and mental state” as more important. Whereas these assessments of more experienced physicians seem to be closer to the patient preferences, the assessments of less experienced physicians might truly reflect a different patient population. However, these findings can also be a misinterpretation of patient preferences due to less patient contacts.

In a second step, preferences were measured using a Discrete Choice Experiment in which the physicians had to choose eight times between two treatment options, which were described by combinations of 6 quality aspects. The influence of each treatment characteristic on the treatment decision was evaluated.

The clearly highest relevance for the treatment selection of physicians as well as patients was found for emotional quality of life indicated by the fact that the disease was not obvious for other persons. From the patient point of view the avoidance of physical impairments such as diarrhea and nausea followed next, and with some distance the possibility of participating in social life – physicians assessed both of these aspects at the same level.

As well as in the previous study on patient assessments of treatment preferences, the DCE results of the current study correspond partly to the direct measurement of needs where emotional, physical and social quality of life were also in the upper range of preferences. Again the characteristics “Maximum increase of life expectancy” and “Avoidance of the risk of long term side-effects” seem to be far less important in DCE than in the direct assessment. This might be due to the fact that the direct assessment using ratings has certain ceiling effects, resulting in all attributes being important. On the contrary the DCE focusses on trade-offs between the attributes. This might result in lower assessments.

As the DCE results show particularly those aspects are weighted high that emphasize HIV/AIDS more as a chronic disease than as directly life threatening condition. As a consequence, therapy adherence of the patients may be increased by better focusing on quality of life arguments than efficacy and safety aspects. However, these arguments differ from results shown in other studies like pill count, dosing frequency and adverse events as shown by Stone et al. [25] or resistance, regimen convenience and sleep disturbance shown by Beusterien et al. [26].

Compared to patients’ preferences concerning the therapy of multiple myeloma [12], the results of the present study demonstrate preference ratings, which may be more affected by the chronic aspect of the disease, whereas preferences of multiple myeloma patients seem to be more affected by the progressive character of their disease leading to increase of life expectancy and possibility of further treatment options as most important factors. Interestingly, in the present study physician assessments of patient preferences were somewhat closer to patient assessments than in multiple myeloma patients. Maybe the preferred recruitment of more experienced physicians in the present study has had an influence and possibly indicates the importance and the need to communicate patient preferences at least to less experienced physicians.

The current study has shown that the preferences of patients and physicians assessing their impression on patients’ preferences were mostly concordant. However, as various studies and a recent review on the concordance of patients and physicians have shown patient preferences and expert judgments can differ [27] and that the results should always be interpreted in the light of actual circumstances given in a study [28].

Some limitations of the current study are to be discussed. Because the DCE is only manageable using a limited number of characteristics and pairs to be compared, decisions were made during the composition of characteristics reflecting the factor structure found in the direct assessment and the construction of comparisons [12]. Doing this, however, some problems remained: dosing aspects were presented as maximum 3 tablets per day versus more, whereas the direct preference measurement revealed more facets of application such as dosing according to current health status, treatment free periods or self-application. As these and some more aspects of application affected at least two factors in the factor analysis, the characteristic “dosing flexibility” may have been defined too simple to reflect the complexity of dosing characteristics. Furthermore, the definition of poles of the characteristics may have influenced the preference decisions: the item “increase of life expectancy” was presented as “increase” versus “maximum increase”. Maybe the perceived difference between these two poles was too small to prefer this characteristic against others. Thus, the somewhat surprising low importance of this efficacy parameter may be the consequence of too close definitions of the poles of this characteristic.

Another limitation arises from the two different samples assed for the comparison. The patient recruitment was performed by the German Competence Network for HIV/AIDS, while the recruitment of physicians was performed using a nationwide database from Janssen-Cilag GmbH. Hence the patients in the previous study are maybe dissimilar from those treated by the doctors in this study. Moreover physicians may have based their assessments on thousands of their patients. These patients might not be similar to the 218 patients in the patient sample.

## Conclusion

In summary, direct preference assessments as well as DCE contribute important findings to the knowledge of AIDS/HIV treatment preferences. On the one hand, these findings cover assessments of patients suffering from AIDS/HIV, which were shown in a previous study [12] as well as the physician judgments. The main result of both studies was the fact that AIDS/HIV-patients as well physicians are congruent in their assessments. There is concordance of both groups that it is very important that an antiretroviral therapy supports the patients’ quality of life, in particular the emotional quality of life. This perhaps reflects a paradigm shift in preferences, since more convenient and less toxic options for cART became available within the last decade.

On the other hand, the understanding of physicians about the patient preferences is important as the selection of a particular treatment regimen should be accompanied by a conscious consideration of features of possible treatment options, which need to be considered in order to maximize patient adherence and compliance to the selected treatment. The results of the current study have shown that the preference assessments of patients themselves and physicians assessing their impression how patients would assess preferences were predominantly in clear concordance. This means that the participating physicians have a good knowledge of the treatment preferences of patients – at least if they are treating or consulting large numbers of AIDS/HIV patients.

This study was based on the presumption: If healthcare services available are tailored to the needs of the target group, it is assumed that the motivation to utilize those services and participate actively in therapy measures can be increased and long-term treatment success improved. The results on treatment preferences demonstrate that the combined use of a direct assessment for rating different levels of importance on one hand and a collection of data by a DCE on the other is reasonable and effective. Both procedures yield comparable results, with the direct assessment being able to cover a greater number of aspects and the DCE focusing on six of the most important aspects.

## Consent

Written informed consent was obtained from the physicians for publication of this report and any accompanying images. The participant had the right and chance to end the study at any time. The decision not to participate or to withdraw from the study did not involve any penalty.

## Competing interests

The research project was financially supported by Janssen-Cilag. JM is employed by Janssen-Cilag. AM, MN and MS declare that they have no competing interests.

## Authors’ contributions

AM and MN designed and carried out the empirical study. MS acted as a clinical adviser and participated in the coordination of the study. JM contributed to the draft of the paper. All authors read and approved the final manuscript.
